# Advances in field-based high-throughput photosynthetic phenotyping

**DOI:** 10.1093/jxb/erac077

**Published:** 2022-02-26

**Authors:** Peng Fu, Christopher M Montes, Matthew H Siebers, Nuria Gomez-Casanovas, Justin M McGrath, Elizabeth A Ainsworth, Carl J Bernacchi

**Affiliations:** 1 Carl R. Woese Institute for Genomic Biology, University of Illinois at Urbana-Champaign, Champaign, IL, USA; 2 Department of Plant Biology, University of Illinois at Urbana-Champaign, Urbana, IL, USA; 3 Department of Crop Sciences, University of Illinois at Urbana-Champaign, Urbana, IL, USA; 4 United States Department of Agriculture, Global Change and Photosynthesis Research Unit, Agricultural Research Service, Urbana, IL, USA; 5 Institute for Sustainability, Energy & Environment, University of Illinois at Urbana-Champaign, Urbana, IL, USA; 6 Center for Advanced Bioenergy and Bioproducts Innovation, University of Illinois at Urbana-Champaign, Urbana, IL, USA; 7 University of Edinburgh, UK

**Keywords:** Field phenotyping, food security, gas exchange, photosynthesis, plant breeding, remote sensing

## Abstract

Gas exchange techniques revolutionized plant research and advanced understanding, including associated fluxes and efficiencies, of photosynthesis, photorespiration, and respiration of plants from cellular to ecosystem scales. These techniques remain the gold standard for inferring photosynthetic rates and underlying physiology/biochemistry, although their utility for high-throughput phenotyping (HTP) of photosynthesis is limited both by the number of gas exchange systems available and the number of personnel available to operate the equipment. Remote sensing techniques have long been used to assess ecosystem productivity at coarse spatial and temporal resolutions, and advances in sensor technology coupled with advanced statistical techniques are expanding remote sensing tools to finer spatial scales and increasing the number and complexity of phenotypes that can be extracted. In this review, we outline the photosynthetic phenotypes of interest to the plant science community and describe the advances in high-throughput techniques to characterize photosynthesis at spatial scales useful to infer treatment or genotypic variation in field-based experiments or breeding trials. We will accomplish this objective by presenting six lessons learned thus far through the development and application of proximal/remote sensing-based measurements and the accompanying statistical analyses. We will conclude by outlining what we perceive as the current limitations, bottlenecks, and opportunities facing HTP of photosynthesis.

## Teaser

High-throughput phenotyping of photosynthesis is critical to continued improvements in crop yield. We review remote and proximal sensing-based phenotyping techniques and outline lessons, challenges, and opportunities facing photosynthesis high-throughput phenotyping.

## Introduction

Photosynthesis accounts for the largest flux associated with the global carbon cycle (Friedlingstein *et al.*, 2019). Photosynthetic rates vary extensively among species and plant functional types; the within-species rates also vary over spatial and temporal scales associated with stage of development and changes in light, temperature, water, and nutrient availabilities (Thornley, 2002; Beer *et al.*, 2010). Photosynthesis is the entry point of carbon into vegetation, and therefore is a critical determinant of food production. Anthropogenic activities are driving global changes, which have profound impacts on all aspects of ecosystem functioning including photosynthetic rates (Fernández-Martínez *et al.*, 2019). A growing population is increasing demands for agricultural products, requiring a doubling of yields by 2050 (Valin *et al.*, 2014). However, current rates of yield improvement fall short of this goal (Ray *et al.*, 2013; Long *et al.*, 2015) and are likely to diminish with continued global climate change. For example, global temperatures and atmospheric CO_2_ concentrations are rising faster than worst-case predictions (Schwalm *et al.*, 2020), and these global changes are shown to strongly influence photosynthetic rates. Warming, regardless of whether from season-long heating (Ruiz-Vera *et al.*, 2013, 2015; Wang *et al.*, 2020) or short duration, high-intensity heat waves (Siebers *et al.*, 2015, 2017; Thomey *et al.*, 2019), has been shown to have a detrimental impact on crop production, even in the presence of an elevated atmospheric CO_2_ concentration.

The need to meet agricultural demands extends from current food shortages in many regions of the planet (Pawlak and Kołodziejczak, 2020) to anticipated future global shortages (Ray *et al.*, 2013; Long *et al.*, 2015). Focused breeding efforts that overcome many of the existing challenges are critical to avoid these food shortages. Improving crop production requires the ability to identify the best varieties for advancement, which have historically included the highest yielding lines, but also a wide range of other phenotypes linked to canopy architecture, lodging tolerance, or protein content. However, these selection criteria are generally measured at physiological maturity or after crop senescence, and do not consider incremental changes in crop phenotype throughout the growing season. While these metrics are responsible for significant advancements in historic crop production (Smith *et al.*, 2014; Specht *et al.*, 2014), the impact of these breeding techniques is diminishing, or has already diminished, entailing the need for new strategies to increase crop productivity. High-throughput phenotyping (HTP) techniques can resolve variation in a wide range of crop traits at shorter time intervals than traditional measurements (Araus and Cairns, 2014; Deery *et al.*, 2014; Bai *et al.*, 2016; Mir *et al.*, 2019; Roitsch *et al.*, 2019; Liu *et al.*, 2020) and can ultimately lead to better understanding of the incremental changes in crop growth and physiology compared with season-integrated composite traits measured after full canopy development or crop harvest.

The mechanistic understanding of photosynthesis is based on decades of measurements at the organelle to plant scales using gas exchange techniques. Key insights from this research have led to the understanding that photosynthesis is inefficient at leaf to canopy scales; for example; the efficiency to convert the intercepted radiation into biomass is only around a fifth of the theoretical maximum for both C_3_ and C_4_ crop species (Zhu *et al.*, 2010). Thus, overcoming these inefficiencies can lead to improved crop yields (Long *et al.*, 2015; Ort *et al.*, 2015). However, measuring photosynthesis over a range of spatial and temporal scales is challenging given the many constraints. Leaf-level measurements using gas exchange techniques are too slow for phenotyping traditional breeding trials even when implementing techniques that rapidly accelerate data collection (Stinziano *et al.*, 2019). Even if throughput of leaf-level measurements is improved, agronomic traits are based on canopy-scale processes and therefore require canopy-scale measurements. Direct measurements of canopy photosynthesis are impractical using enclosures, and therefore micrometeorological, proximal sensing, or remote sensing techniques need to be employed.

In this review, we present high-throughput techniques currently used or in development that estimate photosynthesis from leaf to canopy scales with spectral regions between 350 nm and 2500 nm. Thus, this study will not include a summary of methods in quantification of photosynthesis or photosynthesis-related parameters such as evapotranspiration or stomatal conductance using thermal sensing techniques (or beyond). Neither does this study serve as an exhaustive search of the literature in this field. Following the overview of techniques in HTP of photosynthesis, we outline six lessons learned thus far from the development and application of these techniques, including the use of various sensors, statistical analyses, and limitations. Within each lesson, we outline the current understanding associated with this lesson as well as challenges that must be overcome before widespread adoption is likely for breeders and/or researchers.

## Overview of high-throughput phenotyping techniques for measuring *in situ* photosynthesis and photosynthetic physiology

**Fig. 1.**
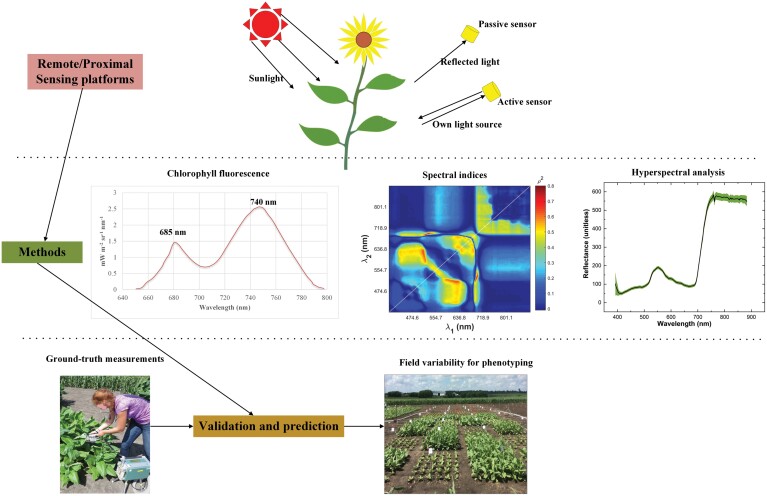
A general overview of remote and proximal sensing techniques used for HTP of photosynthesis. The sensors used in the HTP platforms may be passive or active, dependent on whether these sensors have their own light source. The methods summarized here include those based on chlorophyll fluorescence (either actively or passively measured), spectral indices, and hyperspectral reflectance data. The number in the spectral indices plot represents the squared correlation coefficient between a ratio index and the maximum carboxylation rate, and a higher number indicates a better correlation of such an index with the maximum carboxylation rate. Further details can be found in Fu *et al.* (2020). The reflectance spectra shown here were captured using a hyperspectral camera over a tobacco canopy, and shaded regions show the variability in reflectance spectra within that canopy. The development of remote/proximal sensing methods to estimate photosynthesis requires ground-truth data for both model training and validation.

Despite the benchmark photosynthesis measurements provided by various gas exchange techniques at the leaf level (Long and Bernacchi, 2003; Stinziano *et al.*, 2019), the approach is low throughput (further details can be found in Appendix S1) and has been a bottleneck to the development of crop cultivars with enhanced photosynthesis (Furbank and Tester, 2011; Fu *et al.*, 2019). As such, various HTP platforms have been designed to cope with this low-throughput challenge (Salter *et al.*, 2018; Bai *et al.*, 2019; Bandopadhyay *et al.*, 2020; Meacham-Hensold *et al.*, 2020; Zhang *et al.*, 2020). These platforms, set up in either indoor or outdoor settings, are mounted with commercial sensors such as hyperspectral and fluorescence radiometers, providing a non-invasive and efficient alternative to characterize plant growth and photosynthesis over time. So far, these techniques have had a great impact on understanding of photosynthesis and photosynthetic physiology from leaf to canopy scales, and thus on efforts to improve crop yields through photosynthesis (Siebers *et al.*, 2021). In this section, we provide an overview of proximal/remote sensing techniques used for HTP of photosynthesis/photosynthetic physiology at both leaf and canopy scales (Fig. 1).

### Solar-induced fluorescence

Chlorophyll fluorescence represents light re-emitted by excited chlorophyll molecules and competes with two other pathways, photochemistry and non-photochemical quenching (NPQ), for de-excitation (Porcar-Castell *et al.*, 2014). It has emerged as an important tool to probe the photosynthetic apparatus due to its close and functional linkage with electron transport at the molecular level (Genty *et al.*, 1989; Maxwell and Johnson, 2000). Chlorophyll fluorescence is largely measured in an active way using pulse amplitude modulation (PAM) fluorometry, which can selectively close and open PSII reaction centers to understand the photosynthetic quantum yields of absorbed photons for individual plant leaves (Schreiber *et al.*, 1995). The widespread use of PAM fluorescence for quantifying photosynthesis further stimulates interest to passively detect chlorophyll fluorescence under solar illumination (Troy *et al.*, 2017), known as solar-induced fluorescence (SIF), beyond the leaf scale using remote sensing techniques. Because the SIF signal is small compared with the radiation flux reflected by a plant canopy under sun illumination, SIF is more difficult to retrieve compared with PAM-derived fluorescence. However, great achievements have been made in the development of methods used for decoupling SIF signals from reflected radiance (Meroni *et al.*, 2009; Mohammed *et al.*, 2019). In addition, the increasing availability of SIF imaging (Rascher *et al.*, 2015; Pinto *et al.*, 2016) or sensor systems, such as FluoSpec by Yang *et al.* (2018), also contributes to the popularity of SIF in characterizing plant photosynthesis at various scales.

As improving photosynthesis is considered critical to enhanced crop yield (Long *et al.*, 2015; Ort *et al.*, 2015), SIF has been increasingly used for HTP of photosynthetic physiology (Zavafer *et al.*, 2020). Camino *et al.* (2019) showed that combined SIF and hyperspectral images, obtained through an airborne platform, could be used to estimate the maximum carboxylation rate (*V*_cmax_) for both rainfed and irrigated wheat trials. Using SIF obtained from a ground-based phenotyping platform, Jiang *et al.* (2020) characterized the effective quantum yield of PSII (ϕ_PSII_) and electron transport rate (ETR) for cotton cultivars. In their study, the estimated ϕ_PSII_ was highly correlated to that provided by a PAM fluorometer. Based on time-synchronized hyperspectral and irradiance measurements, Fu *et al.* (2021) derived the inverse relationship between SIF yield and photosynthetic capacity (i.e. *V*_cmax_ and the maximum electron transport rate, *J*_max_) for tobacco cultivars at the canopy level. These studies were stimulated by previous utilization of satellite-based SIF as a proxy of the gross primary productivity (GPP) at ecosystem and global scales (Frankenberg *et al.*, 2011; Guanter *et al.*, 2014; Guan *et al.*, 2016). Unlike satellite-based studies, HTP work aims to detect subtle variations in photosynthetic performance, for example among different crop cultivars, management practices, or environmental conditions. As SIF emissions are largely determined by absorbed photosynthetically active radiation (APAR) (Walther *et al.*, 2016), comparison of SIF and SIF-related parameters from different crop cultivars requires standardization, accounting for plant 2D or 3D architecture in assessing the photosynthetic performance. Combined measurements of SIF and environmental variables, such as temperature and vapor pressure deficit, are also necessary for fully uncovering the functional relationship between SIF and SIF-related parameters and crop photosynthesis.

### Laser-induced fluorescence transient

Compared with passive SIF measurements, active chlorophyll fluorescence observations such as PAM measurements are more commonly used to quantify photosynthetic efficiency, particularly in the context of HTP (Pieruschka *et al.*, 2012). One of the techniques for active chlorophyll fluorescence measurements is laser-induced fluorescence transient (LIFT), which uses subsaturating pulses to probe PSII based on fast repetition rate (FRR) fluorometry (Kolber *et al.*, 1998, 2005). The LIFT system can be operated at a greater distance from the leaf compared with the PAM approach that relies on the application of saturating light flashes in close proximity to photosynthetically active tissue (Genty *et al.*, 1989) for quantifying chlorophyll fluorescence yield. The LIFT approach has demonstrated potential to bridge the gap in photosynthetic measurements between leaf and canopy levels (Raesch *et al.*, 2014; Wyber *et al.*, 2018). Evidence suggests that LIFT-based chlorophyll fluorescence measurements correlate well with PAM-based photosynthetic parameters (Kolber *et al.*, 2005) and can be used to quantify the ETR from the primary quinone acceptor (Q_A_) to the plastoquinone (PQ) pool (Osmond *et al.*, 2017, 2019).

Since the first field observations of laser-induced fluorescence (Measures *et al.*, 1973), new generations of active LIFT fluorometers have been developed and used for plant phenotyping (Ananyev *et al.*, 2005; Kolber *et al.*, 2005; Keller *et al.*, 2019*a*). Keller *et al.* (2019*a*) derived the maximum chlorophyll fluorescence induced by FRR flash and the Q_A_ reoxidation efficiency parameters for phenotyping of photosynthesis from the LIFT-based ETR. Following Keller *et al.* (2019*a*), Keller *et al.* (2019*b*) showed that the LIFT-based parameters could help quantify photosynthetic variations induced by various environment conditions and detect subtle differences in photosynthetic performance among 28 genotypes of four crop species. The operating efficiency of PSII and the kinetics of ETR, as provided through the LIFT approach, can facilitate the assessment of genetic variation in photosynthetic traits in durum wheat under drought conditions (Zendonadi dos Santos *et al.*, 2021). In addition, LIFT fluorometry has also been used onboard airborne sensing platforms, allowing simultaneous assessment of photosynthetic efficiency and GPP (Ounis *et al.*, 2016) for plant phenotyping. These active fluorescence-based measurements enable monitoring of photosynthetic activities at a high temporal resolution regardless of cloud cover conditions.

### Spectral indices

Spectral indices are typically computed using two or more spectral bands, such as red and near-infrared bands, which are highly correlated with vegetation growth and productivity. Because factors such as illumination, atmospheric conditions, and sun sensor viewing geometry can result in large differences in spectral reflectance even for plants of the same species, spectral indices such as the normalized and ratio index are more often used due to their ability to partly remove or even eliminate these observational biases (Myneni and Asrar, 1994). The normalized difference vegetation index (NDVI) (Tucker, 1979) and photochemical reflectance index (PRI) (Gamon *et al.*, 1992) are two exemplar indices derived from satellite images and obtained for characterizing plant photosynthetic performance at the ecosystem level. Compared with the PRI, the NDVI is used as a proxy for vegetation biomass accumulation over time and thus may not be appropriate to quantify short-term variation (e.g. diurnal) of the photosynthetic rate.

In the phenotyping of photosynthesis in field trials, the PRI is probably the most widely used spectral index since it is a proxy of de-epoxidation of the xanthophyll pigments (or the increase of zeaxanthin concentration) (Garbulsky *et al.*, 2011; Peñuelas *et al.*, 2011; Sukhova and Sukhov, 2018) and thus has been connected to NPQ and photosynthetic efficiency (Coops *et al.*, 2010; Goerner *et al.*, 2011). For example, the PRI has been employed as an indicator for assessing the sensitivity of photosynthetic performance in crops to ozone effects (Gray *et al.*, 2010; Ainsworth *et al.*, 2014). However, the correlation of the PRI with NPQ and photosynthetic efficiency is subject to various factors such as illumination intensity, scale (leaf or canopy), and changes in pigments including chlorophyll content and size of the xanthophyll cycle’s pigment pool (Wong and Gamon, 2015; Sukhova and Sukhov, 2018; Yudina *et al.*, 2020). In addition, it remains debated whether the wavelengths used to calculate the PRI (531 nm and 570 nm) at the leaf level are still the best at the canopy scale since light scattering and other confounding effects can induce changes in spectral response of the xanthophyll cycle feature (Garbulsky *et al.*, 2011).

Spectral indices that are related to leaf pigments (e.g. chlorophyll content) and canopy structure have also been used in plant phenotyping of photosynthesis. For example, the structure-insensitive pigment index (SIPI, also known as the chlorophyll index) (Dash and Curran, 2004) has been correlated with the chlorophyll content of vegetation canopies. Since chlorophyll content is one of the important pigments in photosynthesis, the derived chlorophyll content-based index may also be a good indicator of photosynthetic capacity (Croft *et al.*, 2017). Fu *et al.* (2020) used three types of spectral indices including the SIPI, ratio, and NDVI-like indices for estimating photosynthetic capacity with optimized band wavelengths. Their results showed that the squared correlation coefficient (*R*^2^) between spectral indices and photosynthetic capacity can be up to 0.8. However, the relationship between the chlorophyll content and photosynthetic rates may not always hold. as photosynthesis can be influenced by factors such as environmental conditions.

### Hyperspectral analysis

Hyperspectral analysis has become a powerful tool in HTP of photosynthesis and photosynthetic physiology due to its non-destructive nature in sensing of radiance reflected from vegetation. The use of portable hyperspectral radiometers for quantifying photosynthesis is an important step to scale photosynthetic measurements from leaf to canopy levels. Portable hyperspectral radiometers typically have standardized reference panels and radiometrically calibrated light sources. Thus, the relationship between reflectance spectra and the concurrent photosynthetic measurements collected from gas exchange systems can be examined without confounding factors such as leaf scattering and canopy structure. Recent studies suggested that leaf reflectance spectra can be successfully used to estimate key photosynthetic parameters in aspen and cottonwood trees (Serbin *et al.*, 2012), soybean (Ainsworth *et al.*, 2014), wheat (Silva-Perez *et al.*, 2018), maize (Yendrek *et al.*, 2017), and tobacco (Fu *et al.*, 2019). Stimulated by these leaf-level estimations of photosynthetic capacities, hyperspectral imaging (HSI), which can quickly scan hundreds or even thousands of field trials, is being utilized to reveal variability in photosynthetic traits of interest at the canopy level. These HSI sensors can provide data in three dimensions with spectral wavelength across spatial locations, resulting in large amounts of data that need to be analyzed in an innovative way (Siebers *et al.*, 2021).

Approaches linking reflectance spectra to photosynthesis or photosynthetic physiology can be divided into two main categories. The first category refers to the direct correlation of reflectance measurements with photosynthetic measurements (e.g. those derived from gas exchange systems) using machine learning models (Serbin *et al.*, 2012; Fu *et al.*, 2020). These machine learning models, such as partial least square regression (PLSR) (Wold *et al.*, 2001) and least absolute shrinkage and selection operator (LASSO) (Tibshirani, 1996), are used because of their ability to greatly reduce high-dimension hyperspectral data to a few important components or variables. The availability of different machine learning algorithms also provides opportunities to collectively harness the power of these models to estimate photosynthetic physiology, although further examination is necessary to understand the transferability of these algorithms to other species under different environmental conditions (Fu *et al.*, 2019). Recent efforts have also been made toward overcoming the overfitting issue posed by these machine learning algorithms in estimating photosynthesis because of the limited number of training samples (Jin *et al.*, 2022).

The second category in quantifying photosynthetic performance from reflectance spectra refers to methods based on radiative transfer models (RTMs or numerical inversions). RTMs such as PROSAIL (Jacquemoud *et al.*, 2009) and SCOPE (van der Tol *et al.*, 2009) can simulate the movement of photons within vegetation by accounting for canopy biochemical and biophysical characteristics. In the inversion mode, the model input parameters such as chlorophyll content can be varied to yield the best match between observed and simulated reflectance spectra. The best solution to these input parameters is then achieved through iterative optimization of a loss function (Feret *et al.*, 2008). Camino *et al.* (2019) successfully combined SCOPE inversions and SIF to quantify *V*_cmax_ for plant trials under both rainfed and irrigated conditions. Fu *et al.* (2020) showed that RTM-based parameters can explain up to 60% of variance (as demonstrated by the coefficient of determination, *R*^2^) in photosynthetic capacity among 11 tobacco cultivars. Wang *et al.* (2021) suggested that RTM-based chlorophyll and nitrogen contents can well characterize *V*_cmax_ with a correlation coefficient of 0.71. In addition, SIF-oriented RTMs such as Fluospect-B (Vilfan *et al.*, 2016) in the future will play an important role in understanding photosynthetic performance in the context of plant phenotyping. However, these models need to be further examined for their suitability for proximal sensing of plants.

## Lesson 1: traditional remote sensing measurements using multispectral sensors are useful to characterize general ecosystem traits, but lack both the spectral resolution to extract key variables and the precision to capture intraspecific variation in key plant photosynthetic metrics

Much of the work on remote sensing of photosynthesis has been initiated with large-scale satellite-based observations (Siebers *et al.*, 2021) such as Landsat and MODIS images. The era of satellite remote sensing of photosynthesis began in the 1970s when the Earth Resources Technology Satellite 1 (later termed Landsat 1) was launched. The satellite was equipped with a multispectral scanner consisting of four broadband wavelengths including red and near-infrared spectral regions for vegetation observations at 60 m. That is also the era when spectral indices such as the NDVI were designed for characterizing plant biomass and photosynthesis (Tucker, 1979). Since then, a series of multispectral sensors onboard Earth observation satellites with enhanced spectral, spatial, and temporal resolutions have been launched (Table 1). For example, the most recent Landsat satellite is Landsat-9 launched on 27 September 2021, carrying the Operational Land Imager 2 (OLI-2) that has much greater spectral (11 bands) and spatial resolutions (30 m). The MODIS sensors onboard both the Aqua and Terra satellites can scan land surfaces daily at 0.25–0.5 km. The quantification of photosynthesis from remote sensing thus has evolved from simple index-based approaches to those that are based on the synergistic use of remote sensing, flux data (e.g. FLUXNET data), and machine learning (Ryu *et al.*, 2019).

**Table 1.** Major Earth observation satellites for landscape monitoring since the 1970s

Satellite and sensor	Spectral bands (µm)	Spatial resolution (m)	Temporal resolution (days) and data availability	Main applications or variables for vegetation monitoring
Landsat 1–3 multispectral scanner	Band 1: 0.5–0.6 Band 2: 0.6–0.7 Band 3: 0.7–0.8 Band 4: 0.8–1.1	60	16; 1972–1983	Various vegetation indices such as NDVI, PRI; vegetation phenology
Landsat 4–5 thematic mapper	Band 1: 0.45–0.52 Band 2: 0.52–0.60 Band 3: 0.63–0.69 Band 4: 0.76–0.90 Band 5: 1.55–1.75 Band 6: 10.40–12.5 Band 7: 2.08–2.35	Band 6: 120 All other bands: 30	16; 1982–2012	Various vegetation indices such NDVI and PRI; vegetation phenology; land surface temperature
Landsat 7 enhanced thematic mapper plus	Band 1: 0.45–0.52 Band 2: 0.52–0.60 Band 3: 0.63–0.69 Band 4: 0.77–0.90 Band 5: 1.55–1.75 Band 6: 10.40–12.5 Band 7: 2.09–2.35 Band 8: 0.52–0.90	Band 6: 60 Band 8: 18 All other bands: 30	16; 1999–2021	Various vegetation indices such as NDVI and PRI; vegetation phenology; land surface temperature
Landsat 8–9 operational land imager and thermal infrared sensor	Band 1: 0.43–0.45 Band 2: 0.45–0.51 Band 3: 0.53–0.59 Band 4: 0.64–0.67 Band 5: 0.85–0.88 Band 6: 1.57–1.65 Band 7: 2.11–2.29 Band 8: 0.50–0.68 Band 9: 1.36–1.38 Band 10: 10.6–11.19 Band 11: 11.50–12.51	Band 8: 15 Band 10 and 11: 100 All other bands: 30	16; 2013–present	Various vegetation indices such as NDVI and PRI; vegetation phenology; land surface temperature
Terra and Aqua moderate resolution imaging spectrometer	Band 1: 0.62–0.67 Band 2: 0.84–0.87 Band 3: 0.46–0.48 Band 4: 0.55–0.57 Band 5: 1.23–1.25 Band 6: 1.63–1.65 Band 7: 2.10–2.16	Band 1–2: 250 All other bands: 500	Daily; 2000–present	Various vegetation indices such as NIRv, NDVI, and EVI; vegetation phenology; land surface temperature; GPP
Sentinel-2 multispectral imager	Band 1: 0.43–0.45 Band 2: 0.45–0.52 Band 3: 0.54–0.58 Band 4: 0.65–0.68 Band 5: 0.69–0.72 Band 6: 0.73–0.75 Band 7: 0.77–0.79 Band 8: 0.78–0.90 Band 9: 0.93–0.96 Band 10: 1.36–1.39 Band 11: 1.56–1.66 Band 12: 2.10–2.28	Band 1, 9–10: 60 Band 2–4, 8: 10 Band 5–6, 11, 12: 20	~5 d for combined Sentinel-2A and -2B satellites; 2015–present	NDVI, EVI, vegetation phenology

NDVI, normalized difference vegetation index; EVI, enhanced vegetation index; NIRv, the near-infrared reflectance of vegetation is the product of total scene NIR reflectance and the NDVI; GPP, gross primary productivity.

Clearly, traditional satellite remote sensing data (such as those listed in Table 1) are of a coarse spatial resolution that cannot be used to detect any subtle variation in photosynthetic performance in field trials that are typically only a few meters across. As the spectral resolution of these traditional multispectral satellite sensors is typically larger than 20 nm (Table 1), these sensors cannot characterize photosynthetic performance at leaf and canopy levels sufficient for HTP of field trials. This has been evidenced by a recent study showing that the resampling of reflectance spectra to a larger spectral resolution (≥20 nm) can greatly decrease the accuracy in estimating *V*_cmax_ and *J*_max_ (Fu *et al.*, 2020). However, a better understanding of suitability of previous remote sensing methods for characterizing photosynthesis with proximal sensing platforms is needed and can be helpful for application of these sensing techniques in HTP of photosynthesis for field trials.

## Lesson 2: hyperspectral reflectance increasingly shows widespread utility in measuring the physiological controls of photosynthesis

Expanding beyond the coarse-resolution multispectral techniques, recent studies demonstrate that hyperspectral reflectance is a promising tool to measure the biochemical limitations of photosynthesis in both C_3_ and C_4_ species (Table 2). These studies built upon hyperspectral reflectance experiments performed by the remote sensing community that monitored ecosystem-level performance from biophysical relationships of the plant canopy (i.e. canopy greenness, leaf area index, plant architecture, photosynthetic radiation use efficiency, etc.) (Garbulsky *et al.*, 2011). The enthusiasm backing the current wave of hyperspectral reflectance studies of crops at leaf and plot levels is driven by the rapid and data-rich leaf spectra collected by spectroradiometers. The increase in speed over traditional infrared gas analyzer (IRGA) systems for estimating leaf photosynthetic traits is especially beneficial because many more species or genotypes within a species can be measured quite rapidly, enabling studies of genetic diversity (Yendrek *et al.*, 2017). The hyperspectral reflectance captured by many spectroradiometers covers 350–2500 nm (i.e. full range of the spectrum), with various spectral signatures providing information about pigment content, structural components, and water content (Curran, 1989; Gamon *et al.*, 1992, 1997; Peñuelas *et al.*, 1993, 1995). More recently, the full range of spectral data are exploited for understanding plant traits using multivariate modeling and machine learning techniques. This approach has also been used to estimate the biochemical limitations to photosynthesis, namely *V*_cmax_ and *J*_max_ in C_3_ species, and maximum phosphoenolpyruvate (PEP) carboxylase activity (*V*_pmax_) and light- and CO_2_-saturated photosynthesis (*A*_max_) in C_4_ species (Table 2). Provided these hyperspectral reflectance predictive models accurately estimate the desired photosynthetic traits, they can be used to perform high-frequency measurement campaigns to better understand the physiology of the plants over a growing season. These predictive models can also be applied to large populations to better understand the genetic variation and genetic architecture, and possibly select for these photosynthetic traits to improve crop yields in breeding programs (Silva-Perez *et al.*, 2018; Furbank *et al.*, 2021). This new era of hyperspectral reflectance for photosynthetic traits is heavily concentrated on pairing leaf reflectance with gas exchange measurements to build and validate models. A meaningful shift towards developing models and resources that can extract the physiological controls of photosynthesis at the plot level from unmanned aerial vehicle or high-resolution satellite imagery as well as evidence that photosynthetic traits are important in continued yield improvement will probably be needed to see this technique adopted beyond the scientific community.

**Table 2.** Models of photosynthetic capacity developed from leaf-level or canopy-level hyperspectral reflectance measurements

Reference	Species (organized by trees and crops)	Scale	Modelling approach	Initial slope *R*^2^, RMSE	*J*_max_ *R* ^2^, RMSE	*A*_max_ *R* ^2^, RMSE
	**Trees**					
Doughty *et al.* (2011)	Tropical tree and palm (mixed species)	Leaf Canopy	PLSR			0.47, 5.1 0.49, 4.7
Serbin *et al.* (2012)	*Populus tremuloides*, *P. deltoides*	Leaf	PLSR	0.89, 15.4	0.93, 18.7	
Dechant *et al.* (2017)	*Morus alba*, *Prunus serotina*, and 35 additional tree species	Leaf	PLSR	0.64, 17.36	0.70, 27.77	
Barnes *et al.* (2017)	*Populous deltoids*	Leaf	PLSR	0.72, 4.2	0.72, 18.2	
J. Wu *et al.* (2019)	Tropical tree (mixed species)	Leaf	PLSR	0.89, 6.6		
Jin *et al.* (2020)	Temperate tree (mixed species)	Leaf	SI	0.50, NA	0.67, NA	
Calzone *et al.* (2021)	*Punica granatum*	Leaf	PLSR			0.73, 0.76
Jin *et al.* (2022) ^*a*^	Temperate tree (mixed species)	Leaf	PLSR	0.69, 0.2	0.87, 0.15	
Lamour *et al.* (2021)	Tropical (mixed species)	Leaf	PLSR	0.74, 13.1	0.73, 19.8	
Yan *et al.* (2021)	Temperate, subtropical, tropical (mixed species)	Leaf	PLSR	0.77, 9.7		
Zhou *et al.* (2021)	*Citrus limon*	Leaf	RF, SVM, GDboost, Adaboost			0.64–0.92, 1.84–2.55
	**Crops**					
Ainsworth *et al.* (2014)	*Glycine max*	Leaf	PLSR	0.88, 13.4		
Serbin *et al.* (2015)	Nine California cropping systems	Canopy	PLSR	0.94, 11.56		
Heckmann *et al.* (2017) ^*a*^	*Brassica oleracea* *Zea mays* *Moricandia* (mixed species)	Leaf	PLSR, NN	0.6, 0.016 0.58, 0.013 0.65, 0.019		0.51, 3.99 0.69, 3.38 0.44, 4.89
Yendrek *et al.* (2017)	*Zea mays*	Leaf	PLSR	0.43, 20.64		0.65, 6.6
Silva-Perez *et al.* (2018)	*Triticum aestivum*	Leaf	PLSR	0.62, 20.68	0.7, 25.54	
Fu *et al.* (2019)	*Nicotiana tabacum*	Leaf	PLSR, NN, SVM, LASSO, RF, GP	0.60-0.65, 41.7-54.0	0.45–0.56, 40.1–44.7	
Meacham-Hensold *et al.* (2019)	*Nicotiana tabacum*	Leaf	PLSR	0.77, 10.83	0.72, 10.76	
Meacham-Hensold *et al.* (2020)	*Nicotiana tabacum*	Canopy	PLSR	0.79, 11.9	0.59, 11.5	0.54, 10.6
Cotrozzi *et al.* (2020)	*Zea mays*	Leaf	PLSR			0.86, 6.93
Fu *et al.* (2020)	*Nicotiana tabacum*	Canopy	PLSR, RTM, SI	0.78–0.84, 33.8–38.6	0.80–0.81, 22.6–23.4	
Kumagai *et al.* (2021)	*Glycine max*	Leaf	PLSR, RR, LASSO, SVR	0.57–0.65, NA	0.48–0.58, NA	
Sexton *et al.* (2021)	*Nicotiana tabacum*	Leaf	PLSR	0.81, 18.1		0.86, NA
Wang *et al.* (2021)	*Zea mays*	Leaf	PLSR, RTM			0.66, NA

Reported traits include the initial slope derived from *A*/*C*_i_ curves (Rubisco maximum carboxylation capacity, *V*_cmax_ in C_3_ plants and maximum PEP carboxylase activity, *V*_pmax_, in C_4_ plants), and maximum electron transport capacity (*J*_max_) in C_3_ species, and light- and/or CO_2_-saturated photosynthesis (*A*_max_). For each trait, the goodness of fit for the predictive model (*R*^2^) and the root mean square error (RMSE) are reported. When multiple PLSR models were presented in a given publication, a single model was selected for the table. When multiple machine learning approaches were provided, the range of model fits is provided. Abbreviations: partial least squares regression (PLSR), development of new spectral (vegetation) indices or use of indices in new models (SI), radiative transfer model (RTM), neural network (NN), support vector machine (SVM), least absolute shrinkage and selection operator (LASSO), random forest (RF), Gaussian process (GP), gradient boost (GDboost), adaptive boosting (Adaboost). Further summary of additional information and context for studies listed in Table 2 can be found in Appendix S1.

^*a*^ A normalized RMSE.

## Lesson 3: it is not yet clear whether high-throughput phenotyping techniques have the precision needed to infer small changes in photosynthesis

One of the potential benefits of using leaf reflectance to predict photosynthetic capacity is the ability to analyze thousands of different crop genotypes for quantitative genetic studies. This is impractical with gas exchange techniques because of the time required to make measurements (Grzybowski *et al.*, 2021). However, it is not yet clear that HTP techniques have the same precision as IRGAs to detect small differences in photosynthetic traits within a mapping population. Moreover, IRGAs enable tight regulation of the environmental conditions (e.g. light intensity, relative humidity, CO_2_ concentration, and temperature) surrounding the leaf so that multiple genotypes can be measured and compared under the same environment. Variation in environmental conditions in nature can have a greater effect on photosynthesis than genotype (Kumagai *et al.*, 2022), so HTP techniques for testing genetic variation in photosynthesis need to minimize the influence of environmental variation. Even with these challenges, studies have estimated photosynthetic capacity in diverse populations using hyperspectral reflectance (Yendrek *et al.*, 2017; Silva-Perez *et al.*, 2018). Furbank *et al.* (2021) further created a Web application for wheat breeders to upload hyperspectral reflectance measurements and then receive predicted photosynthetic traits. This tool will enable a community effort to study variation in photosynthetic traits among wheat genotypes, which would improve the precision for detecting small differences in photosynthetic capacity within species.

It is important to consider which statistical metrics can be used to compare the accuracy of different types of machine learning approaches for trait estimation and to determine the accuracy of HTP techniques compared with ‘gold standard’ approaches (i.e. gas exchange techniques). There have been reviews of hyperspectral studies that use the *R*^2^ and the root mean square error of predictive models to compare the quality of models between experiments (e.g. Grzybowski *et al.*, 2021). While these terms are useful for explaining the proportion of the variance for a dependent variable that is explained by independent variables in a regression and provide a measure of the spread of residuals, these metrics are not necessary appropriate tools for comparing the quality of different methods (Martin Bland and Altman, 1986).

To assess the quality of a HTP technique, other practical benchmarks might be more informative. For example, if the objective of using a HTP technique is genetic analysis, one could consider if the same loci and estimates of heritability are apparent with a standard versus HTP technique. Recently, Choquette *et al.* (2019) found that photosynthetic capacity estimated from hyperspectral techniques had a lower heritability than direct measures of photosynthesis using a gas exchange analyzer. In another study, Zendonadi dos Santos *et al.* (2021) found that LIFT techniques detected chlorophyll fluorescence differences between durum genotypes which may be strong enough to use for genome-wide association study (GWAS) analysis. Greater efforts are needed to make multiple repeated measurements of the same plot to demonstrate the limitations of different HTP techniques and methods. Variation in growing season conditions or differences in phenology within a season can also cause changes to photosynthetic capacity and are important to detect (Kumagai *et al.*, 2022). Year-to-year variation decreases the effectiveness of specific PLSR models to predict photosynthesis from reflectance (Ge *et al.*, 2019; Meacham-Hensold *et al.*, 2019), and thus more research is needed to fully evaluate the transferability of models (Grzybowski *et al.*, 2021); that is, whether models developed based on data from one set of field plots/trials can be applied to another set of field plots/trials.

## Lesson 4: scalability of high-throughput phenotyping techniques is uncertain

The scalability of high-throughput techniques for measuring photosynthetic traits is an open question for researchers and is a necessary consideration before methods are more broadly adopted. As shown in Table 2, most hyperspectral reflectance models predicting physiological constraints of photosynthesis are based on leaf spectra collected at the leaf surface, which are then correlated to gas exchange measurements. However, it is unclear how predictions of photosynthetic performance scale spatially from leaf to canopy scale using measurements such as those from drone- and airborne-based platforms that are critical to phenotyping of photosynthesis in a large number of field trials (Araus and Cairns, 2014).

**Fig. 2.**
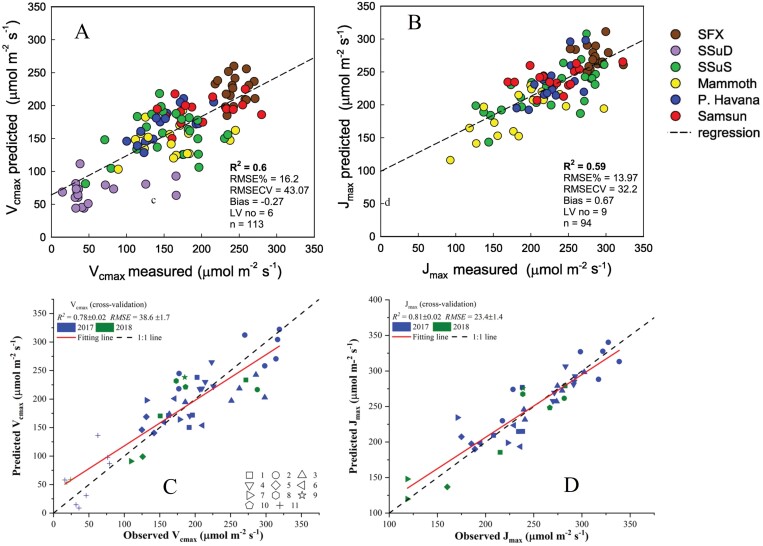
*V*_cmax_ and *J*_max_ predictions at leaf (A and B) and canopy (C and D) scales for the same field trials. All predictions were made using the PLSR method with inputs of reflectance spectra collected using portal spectroradiometers (A and B) and hyperspectral imaging (C and D) for all tobacco cultivars on different dates. The colors in (A and B) and shapes in (C and D) represent different tobacco cultivars. This figure was adapted from Meacham-Hensold *et al.* (2019) and Fu *et al.* (2020), and further details related to the PLSR modeling can be found in these two studies. The better prediction performance at the canopy level may be attributed to the spatial averaging of photosynthetic parameters (*V*_cmax_ and *J*_max_) and pixel-based reflectance spectra which partly removed intraplot variations that can be seen from leaf-level analysis.

Even with the increased speed in collecting data of spectroradiometers compared with portable gas exchange systems, researchers performing surveys with direct leaf sampling attachments are limited in their ability to capture leaf reflectance data from more than a couple of leaves per plot in a timely manner. Other proximal HTP techniques have similar time constraints. Plot-level measurements of chlorophyll fluorescence were captured on ~220 accessions of wheat using LIFT (Zendonadi dos Santos *et al.*, 2021). Those data were collected at an average speed of 8 cm s^–1^. Canopy-level hyperspectral measurements can take 1–2 min if the cameras need to rotate to scan the field trials (Meacham-Hensold *et al.*, 2020). The drone- and airborne-based sensing platforms can help relieve the time constraints (Camino *et al.*, 2019; Suarez *et al.*, 2021) but may have payload limitations that need to be resolved. Additionally, as canopy-level measurements are scaled up, a large volume of data can be expected (Sagan *et al.*, 2021) and pose difficulties to manage and process (Fu *et al.*, 2020; Meacham-Hensold *et al.*, 2020). Large differences are also observed in models built using leaf-level hyperspectral reflectance and those using canopy-level hyperspectral imaging for the same field trials (Fig. 2) since leaf-level and canopy-level reflectance spectra are not necessarily identical, making direct comparisons difficult (Meacham-Hensold *et al.*, 2019; Fu *et al.*, 2020). Thus, further efforts are needed to understand what factors and processes lead to the variability of models from leaf to canopy levels.

## Lesson 5: data and methods require standardization so that sound inferences can be made across time, space, and species

Plant phenotyping generates a large amount of data, and processing these data is complex (Cobb *et al.*, 2013; Coppens *et al.*, 2017; Fu *et al.*, 2019, 2020). With current advances of various HTP techniques for measuring photosynthetic traits, standardizing image data collection, processing, and analysis are crucial so proper inferences can be made (Araus and Cairns, 2014; Shakoor *et al.*, 2019; Li *et al.*, 2021). Yet, imaging devices, computer-vision techniques, and software packages are abundant, and obtaining a unified and robust suite of standard tools and protocols remains a challenge (Fahlgren *et al.*, 2015; Song *et al.*, 2021). Despite this challenge, recent advances in the creation of guidelines for best practices for data acquisition, open-source image analysis tools, and automated image analysis pipelines are becoming more and more common (Gehan *et al.*, 2017; Berry *et al.*, 2018; Burnett *et al.*, 2021). For example, Burnett *et al.* (2021) presented a practical guide and a free tutorial for breeders and researchers on the use of the PLSR modeling method that allows the prediction of physiological traits from leaf-level hyperspectral data including *V*_cmax_ and *J*_max_.

Central to advance the field of phenotyping is access to datasets for the identification of novel and potentially new interesting results that can further provide the foundation on which different data streams can be used to inform models (Danilevicz *et al.*, 2021). This is because one of the most time-consuming and costly aspects of HTP is the correlation of traits to measured physiological processes, and many studies generally focus on specific questions despite there being more information that can be extracted from phenotyping datasets using different or new approaches and techniques (Fiorani and Schurr, 2013; Singh *et al.*, 2016; Ubbens and Stavness, 2017). However, many publications do not provide the needed accessible metadata (e.g. extensive description of data collection methodology, biological information, and experimental conditions), raw data, and code source for further analyses (Rosenqvist *et al.*, 2019). In the face of this challenge, many journals and funding agencies are now requiring researchers to store and give access to this information in open access repositories and libraries. The NASA-funded Ecological Spectral Information System (EcoSIS, www.ecosis.org) and Ecological Spectral Model Library (https://www.ecosml.org) represent examples of a database and library designed to store spectral and ancillary measurements as well as model codes. Currently, the EcoSIS spectral library contains 172 datasets (Wagner *et al.*, 2018), and the accumulation of publicly available data and model code will not only help identify areas for computation tool improvements but also accelerate multispecies, multiyear, and cross-site comparisons for meaningful insights to enhance photosynthesis and crop productivity under varied environmental conditions.

## Lesson 6: for a single high-throughput phenotyping trait, it is not clear whether one model can be applied to multiple species within a functional group or, ideally, for all species in general

Approaches to predict photosynthetic capacity from spectra rely on statistical methods that do not necessarily produce accurate predictions when input spectra are outside of the range of the training data (Meacham-Hensold *et al.*, 2019). This raises the question of whether a single method can be developed that will work for all individuals within a species or for all species in general. For example, if a compound unrelated to photosynthetic capacity, but which absorbs in similar wavelengths, exists in one species and not another, a model parameterized with only one species may incorrectly predict capacity of the other. However, if there are differences between the absorption spectra of the photosynthesis-related and unrelated compounds, with a large variety of values, a model could be developed that accounts for these effects. It is reasonable then that a potential solution for a globally applicable model is to collect data and build a model using a large range of species and genotypes within species (Serbin *et al.*, 2015).

Building such a model is challenging and so an alternative, simpler approach would be attractive. Similar methods that relate spectra to physiological parameters are applicable across species, such as chlorophyll fluorescence and the PRI (Rascher *et al.*, 2007). Examining *in vitro* spectrophotometric methods to measure quantities related to *V*_cmax_ and *J*_max_, Rubisco activity, and chlorophyll concentration provides insights into how hyperspectral methods could be adapted to work for many species. In contrast to machine learning methods such as PLSR, which are treated as a black box, methods based on chlorophyll fluorescence and the PRI rely on mechanistic understanding of the relationship between the processes of interest and wavelengths used in the calculations.

*In vitro* spectrophotometric methods to measure Rubisco activity rely on spectral properties of compounds other than Rubisco, for example NADH (Scales *et al.*, 2014),. In this case, the mechanistic understanding of the process is used to isolate measurement to a single, easily measured compound. However, since many processes affect NADH concentration *in vivo*, this mechanism probably cannot be used to develop an *in vivo* hyperspectral method to estimate *V*_cmax_. Potentially a mechanistic model for *J*_max_ is more tractable as chlorophyll is easily measured spectrophotometrically and its concentration is potentially a key limit to *J*_max_. As such, measuring chlorophyll concentration itself *in vivo* in a generally applicable way seems promising. However, chlorophyll is only one limitation to *J*_max_ and, given the numerous other limitations, a mechanistic approach using only spectrophotometry seems unlikely. Recent advances in measurements of SIF, which is a function of light absorption by chlorophyll and the functioning of photosynthetic electron transport, demonstrates its ability to infer *J*_max_ (Fu *et al.*, 2020). However, to extract both *V*_cmax_ and *J*_max_ from high-throughput measurements, the most promising outlook for a universal (or near universal) method may require a model built from a comprehensive dataset.

Given the large number of crop species and the variety of compounds that absorb, reflect, or fluoresce, developing a universal model would be difficult and time consuming. As with other large-scale endeavors, researcher-based networks such as the EcoSIS (www.ecosis.org) are being developed to share datasets useful for building and training models. Considering the variety of equipment, which can vary in spectral resolution, range of wavelengths, and sensitivity, and other experimental considerations such as temperature and light source, standardized documentation or protocols would help ensure that individual datasets can be combined for model development (as discussed in Lesson 5). As these datasets are collected from different plant species/functional types, it would be a good starting point to build predictive models by plant species or functional types, for which further studies are warranted.

## Outlook for high-throughput phenotyping of photosynthesis

Ideally, HTP techniques would be low cost, require little training and expertise to use, provide precise measurements, and reliably operate for hundreds of hours of use. Costs of the HTP sensors for phenotyping photosynthesis are high, but within the same order of magnitude as traditional instruments (e.g. gas exchange systems). Some manufacturers produce equipment that is reliable and easily collects spectra in the field, but other systems may not be well adapted for field use and require substantial in-house development to adapt for field use. Furthermore, most systems allow for relatively easy data collection but the development and use of models to extract photosynthetic traits require in-depth technical expertise. The requirement for in-depth technical expertise thus limits initial users of these methods to advanced breeders and experimental researchers. For these groups, the expense and in-house adaptations may be acceptable costs for using the equipment, but the technical challenge to develop and use these models will probably remain a large barrier to adoption. For widespread use, the equipment is likely to need integrated and pre-developed models (Furbank *et al.*, 2021) so that users can easily collect data and have the instrument output-derived data, similar to advanced gas exchange systems.

Beyond scientific applications, it is unlikely that farmers would adopt this technology without it providing a clear way to improve yield. One approach might be to use these techniques to identify regions of fields with low photosynthetic capacity and then determine factors and/or variables associated with the low photosynthetic capacity. These problem areas could then be addressed by the farmer or land manager as needed for improved crop production. In the major agricultural regions, this may be of little use since the scaling of HTP platforms and techniques to a large scale is not a trivial task. For regions where excess applications are cost prohibitive, the expense of HTP equipment may also be cost prohibitive but, as these techniques advance, the price may become more affordable, ease of use improved, and data outputs easier to obtain. As improving photosynthesis is considered one of the potential strategies for increased crop production to meet rising food demands (Long *et al.*, 2015; Ort *et al.*, 2015; A. Wu *et al.*, 2019), the main benefit of HTP techniques for photosynthesis phenotyping would be to help provide more efficient, resilient, and productive crop cultivars to farmers.

## Supplementary data

The following supplementary data are available at *JXB* online. 

Appendix S1. Further details related to gas exchange measurements for photosynthesis phenotyping and additional information related to studies listed in Table 2. 

erac077_suppl_Supplementary_Appendix_S1
