# Supplementary material for: Advances in field-based high-throughput photosynthetic phenotyping
Source: J Exp Bot. 2022 Feb 26;73(10):3157–72. doi: 10.1093/jxb/erac077 (PMC9126737; doi:10.1093/jxb/erac077)
Supplement: erac077_suppl_Supplementary_Appendix_S1 [file erac077_suppl_supplementary_appendix_s1.pdf]

Appendix S1

This section provides further details related to gas exchange measurements for photosynthesis phenotyping and additional information related to studies listed in Table 2 in the publication.

1 Summary of gas exchange measurements

It has been mentioned that measurements from traditional techniques such as infrared gas analyzer (IRGA) can be too slow for practical use in a breeding trial. Estimating common photosynthetic traits such as V_{cmax} or J_{max} using HTP techniques can be 60 to 100 times faster. Transporting an IRGA between plots, selecting a leaf, and clamping the cuvette onto the leaf takes one to two minutes. Allowing the leaf to acclimate to the chamber conditions takes another one to five minutes. A "bad" leaf or one that may not be light acclimated would require the selection of an entirely different leaf. Running default, A vs. C_i curves on a LiCOR-6800 takes about 12-15 minutes. In all, that is 20 to 30 minutes per curve and the time increases if the canopy is taller, like corn, miscanthus or sorghum, or if the plots are spaced further apart. Canopy level hyperspectral estimates of V_{cmax} or J_{max} requires one to five minutes to set the proper exposure time for the cameras and then 5 to 10 seconds per plot. References need to be included in each measurement. Handheld hyperspectral measurements take 30-40 seconds within each plot. If one wants to screen a field trial consisting of a thousand of soybean genotypes large soybean population for V_{cmax} or J_{max} - assuming measurements are taken between 10 am and 2pm - it would take four LiCOR 6800 25 days to measure all plots. Canopy level estimates of the same populations that size using hyperspectral cameras can be made in a single day if there is constant light.

2 Summary of additional information and context for studies listed in Table 2.

This supplemental summary is to provide additional information and context pertaining to the materials and environment(s) for studies listed in Table 2. Most of the studies listed in Table 2 attempt to capture trait space of the phenotype under observation to build a predictive model involving linear or nonlinear (machine learning) models. This is often done by leveraging diversity across plant ecotypes or genetic diversity within a given species, sampling at different leaf development stages or light levels (sunlit or shaded), in different experimental treatments to induce stress, across multiple years or between *in situ* and greenhouse grown plants.

Doughty *et al.* (2011)– Leaf spectroscopy and tissue sampling were conducted in two different years and multiple locations. Sunlit, intact leaves were sampled on Hawaii Island in multiple locations in January and April with varying rainfall (2000-4000mm). Samples were also taken from the tropical rainforest biome and the display center of Biosphere 2 located near Tucson, AZ, USA where the average air temperature was ~25°C and humidity of ~65%.

Serbin *et al.* (2012) – Greenhouse-grown aspen and cottonwood seedlings were treated to three different temperature regimes with day/night air temperatures (°C) as follows 30/23, 25/18, 20/13 that were used for paired leaf reflectance and photosynthetic. This temperature range was chosen to simulate the air temperatures observed in a latitudinal transect limiting the geographic distributions of temperate and boreal tree species. The authors tested the viability of the

greenhouse produced model on paired leaf reflectance and gas exchange data from data collected *in situ*.

Dechant *et al.* (2017) – The authors conducted their experiment at The University of Leipzig's Arboretum in Central Germany with deciduous and coniferous tree species sampled during daylight hours (9 – 19h) in August and early September. The authors attempted to maximize the number of species and the light environments (sun and shade leaves) of the paired leaf reflectance and gas exchange estimates.

Barnes *et al.* (2017)– The study period took place from late May to early July in 2016 near the Biosphere 2 Research Center, AZ, USA. The goal was to determine if reflectance based high-throughput modelling could be used to detect temporal changes in photosynthetic capacity. The average high temperature during the experiment was 34.4°C and the average low was 21.3°C. Irrigation was varied to simulate different leaf water potentials.

Wu *et al.* (2019) – Two locations in Panama, Parque Natural Metropolitano and San Lorenzo Protected Area, were used for data collection with mean annual temperature of 26°C for both locations and precipitation of 1826 and 3286 mm yr^{-1}. An additional location in Brazil in the Tapajos National Forest was also used with a mean annual temperature of 26°C and precipitation of 2022 mm yr^{-1}. All three locations have a pronounced dry period. The authors concentrated on differences in leaf age and light environments with all measurements being conducted during the dry season.

Jin *et al.* (2020) and Jin *et al.* (2021) – The study location is in a forestry research facility associated with the Shizuoka University, Shizuoka Prefecture, Japan. The forest is a deciduous temperate forest with elevation variation between 390 – 1560 m and annual precipitation of 2153 mm. Multiple tree species were sampled across multiple years with both sun and shaded leaves used in model development.

Calzone *et al.* (2021) – Two commercial pomegranate cultivars that were two-year-old potted plants at the time of the experiment were grown at the San Piero a Grado field-station (Pisa, Italy) in a greenhouse setting. The day/night mean temperatures were 27/21°C for the duration of the short experiment. The primary aim of this experiment was to determine salt stress based on leaf reflectance signatures.

Lamour *et al.* (2021)– The authors built on the data presented in Wu *et al*. (2019) and also collected paired sample data from the San Lorenzo Protected Area in Panama. Please see for more information about the specific environmental conditions above. Multiple species, canopy positions, and leaf ages were used in the development of the predictive models.

Yan *et al.* (2021)– Three diverse forest sites representing vastly different ecosystems in China were sampled for this experiment. These were selected because they represent temperate (Mountain Changbai, CB), subtropical (Mountain Dinghu, DH), and tropical (Xishuangbanna, XSBN) with dramatically different temperatures and annual precipitation (CB: 2.8°C, 691 mm yr^{-1}, DH: 20.9°C 1927 mm yr^{-1}, and XSBN: 21.8°C, 1493 mm yr^{-1}). The authors collected pair leaf reflectance data of the dominant tree species in the given location.

Zhou *et al.* (2021) – *Citrus limon* was used to investigate the ability to detect water stress and photosynthetic capacity in a greenhouse setting in Wuhan, Hubei Province, China. The authors report the regional weather, but because this experiment was conducted in the greenhouse, we are not sharing that information. The authors limited water for plants to induce stress and measured leaves at the top, middle, and bottom of the small, potted trees.

Ainsworth *et al.* (2014)– Two soybean cultivars grown in the field under ambient and elevated (~100 ppb) ozone concentrations in 2011 with 0.76m row spacing were used in the development of the PLSR models shown in this experiment. The project took place at the Soybean Free Air Concentration (SoyFACE) facility located in Champaign, IL, USA. Upper canopy, fully developed and sunlit leaves were used with the primary variation being captured in the experiment being the ozone differences, but no other environmental information was provided.

Serbin *et al.* (2015) – The experiment was conducted in the in the spring and early summer in 2013 and 2014 across three difference research stations in southern California (Kearney Agricultural Extension and Research Center – Parlier, CA, South Coast Research and Extension Center – Irvine, CA, and Coachella Valley Agricultural Research Station – Thermal, CA). 9 crops species across 13 different agroecosystems were sampled over the two years of the study where leaf level measurements were paired with AVRIS data.

Heckmann *et al.* (2017) – In the summer of 2015, plants growing in greenhouse and in the Botanical Garden at the Heinrich-Heine University, Dusseldorf, Germany, were used in the study. Special concentration was given to a Brassica oleracea and Zea mays in the greenhouse across two years with different nitrogen fertilizer applications, a low-N and sufficient-N. No environmental information is provided, but the plants were investigated ~8-10 weeks after germination.

Yendrek *et al.* (2017)– Across three years, the authors grew more than 100 inbred and hybrid maize genotypes in 0.76m row spacing at the SoyFACE Research Facility under ambient and elevated ozone concentrations (~100 ppb). No environmental data is provided for the field part of the experiment. Paired leaf reflectance and gas exchange traits from the field grown maize experiment was also combined with a nitrogen treatment experiment in which a single maize inbred line was grown in the greenhouse during one year of the experiment. Natural light was supplemented with metal halide lamps to provide a minimum photosynthetic photon flux density of 200 umol m^{-2} s^{-1} from 6-20 hr. Paired measurements across both the field and greenhouse experiments were used to estimate photosynthetic leaf traits with leaf reflectance.

Silva-Perez *et al.* (2018)– Five experiments were conducted in greenhouses and in the field in Australia (CSIRO Black Mountain – greenhouse, and GES-CSIRO – field) and Mexico (CENEB-CIMMYT – field) using diverse wheat germplasm. Paired sampling occurred at different development stages both before and following anthesis. Low and high nitrogen treatments were also implemented in the greenhouse experiments.

Fu *et al.* (2019) and Meacham-Hensold *et al.* (2019)– Six varieties of tobacco with contrasting photosynthetic capacity were grown over two field seasons. The papers showed that the high

throughput methods contained within allowed for estimations of these traits even in transgenic plants. The same research materials were used, but the statistical approaches differed between the works.

Fu *et al.* (2020) and Meacham-Hensold *et al.* (2020)– Ten different varieties of wild type and transgenic tobacco were grown in the field with the goal of developing methods of accurately estimating leaf level traits from canopy hyperspectral imaging. Paired reflectance and measured data were collected at different times during the field season to attempt to capture differences in development.

Cotrozzi *et al.* (2020) – The authors built PLSR models predicting hybrid maize phenotypes using facilities located at Purdue University's (West Lafayette, IN, USA) greenhouse (Horticulture Plant Growth Facility) and fields (Agronomy Center for Research and Education). The greenhouse experiment imposed a drought treatment on the hybrid maize varieties and the day/night temperatures were 26/20°C with relative humidity between 50-60%. Standard agronomic practices for the area were used for the field experiment with paired samples being taken from 11-16hr. The authors report 257.53mm of precipitation and the average, maximum, and minimum temperatures (22.7°C, 27.6°C, and 16.3°C) over the duration of the data collection.
Kumagai *et al.* (2021) – The authors collected paired leaf reflectance and photosynthetic traits across two field seasons where they augmented the canopy temperature using infrared heating elements in the field with the goal of increasing the canopy temperature above ambient (ambient, +1.5, +3, +4.5, +6°C). The goal was to determine if high-throughput techniques could be used to identify photosynthetic differences in temperature across the duration of the growing season. Average July and August air temperatures in 2018 and 2019 were 23.2°C and 23.5°C with the reported precipitation being 240 and 146.8 mm.

Sexton *et al.* (2021) – Growth chamber grown wild type and transgenic tobacco plants were grown at high CO_2 (3000 umol mol^{-1}), 75%RH, 12h diurnal cycle with day/night temperatures set to 26°C/22°C. The light level of the chambers were set to 1000umol photons m^{-2} s^{-1} and the paired reflectance and photosynthetic traits were collected between 4-6 weeks after planting.

Wang *et al.* (2021) – Data was collated from Yendrek *et al*. (2017) and collected an independent set of maize grown in the field (Champaign, IL) at different nitrogen treatments and different nitrogen application methods was also sampled of paired leaf reflectance and photosynthetic data. Plants were also sampled at different vegetative stages.

Reference

Ainsworth EA, Serbin SP, Skoneczka JA, Townsend PA. 2014. Using leaf optical properties to detect ozone effects on foliar biochemistry. Photosynthesis Research **119**, 65-76.
Barnes ML, Breshears DD, Law DJ, van Leeuwen WJD, Monson RK, Fojtik AC, Barron-Gafford GA, Moore DJP. 2017. Beyond greenness: Detecting temporal changes in photosynthetic capacity with hyperspectral reflectance data. PLOS ONE **12**, e0189539.

Calzone A, Cotrozzi L, Lorenzini G, Nali C, Pellegrini E. 2021. Hyperspectral Detection and Monitoring of Salt Stress in Pomegranate Cultivars. Agronomy **11**, 1038.
Cotrozzi L, Peron R, Tuinstra MR, Mickelbart MV, Couture JJ. 2020. Spectral Phenotyping of Physiological and Anatomical Leaf Traits Related with Maize Water Status. Plant Physiology **184**, 1363-1377.
Dechant B, Cuntz M, Vohland M, Schulz E, Doktor D. 2017. Estimation of photosynthesis traits from leaf reflectance spectra: Correlation to nitrogen content as the dominant mechanism. Remote Sensing of Environment **196**, 279-292.
Doughty CE, Asner GP, Martin RE. 2011. Predicting tropical plant physiology from leaf and canopy spectroscopy. Oecologia **165**, 289-299.
Fu P, Meacham-Hensold K, Guan K, Bernacchi CJ. 2019. Hyperspectral Leaf Reflectance as Proxy for Photosynthetic Capacities: An Ensemble Approach Based on Multiple Machine Learning Algorithms. Frontiers in Plant Science **10**, 730.
Fu P, Meacham-Hensold K, Guan K, Wu J, Bernacchi C. 2020. Estimating photosynthetic traits from reflectance spectra: A synthesis of spectral indices, numerical inversion, and partial least square regression. Plant, Cell & Environment **43**, 1241-1258.
Heckmann D, Schlüter U, Weber APM. 2017. Machine Learning Techniques for Predicting Crop Photosynthetic Capacity from Leaf Reflectance Spectra. Molecular Plant **10**, 878-890.
Jin J, Arief Pratama B, Wang Q. 2020. Tracing Leaf Photosynthetic Parameters Using Hyperspectral Indices in an Alpine Deciduous Forest. Remote Sensing **12**, 1124.
Jin J, Wang Q, Song G. 2021. Selecting informative bands for partial least squares regressions improves their goodness-of-fits to estimate leaf photosynthetic parameters from hyperspectral data. Photosynthesis Research, online only, https://doi.org/10.1007/s11120-11021-00873-11129.
Kumagai E, Burroughs CH, Pederson TL, Montes CM, Peng B, Kimm H, Guan K, Ainsworth EA, Bernacchi CJ. 2021. Predicting biochemical acclimation of leaf photosynthesis in soybean under in-field canopy warming using hyperspectral reflectance. Plant, Cell & Environment, https://doi.org/10.1111/pce.14204.
Lamour J, Davidson KJ, Ely KS, Anderson JA, Rogers A, Wu J, Serbin SP. 2021. Rapid estimation of photosynthetic leaf traits of tropical plants in diverse environmental conditions using reflectance spectroscopy. PLOS ONE **16**, e0258791.
Meacham-Hensold K, Fu P, Wu J, Serbin S, Montes CM, Ainsworth E, Guan K, Dracup E, Pederson T, Driever S, Bernacchi C. 2020. Plot-level rapid screening for photosynthetic parameters using proximal hyperspectral imaging. Journal of Experimental Botany **71**, 2312-2328.
Meacham-Hensold K, Montes CM, Wu J, Guan K, Fu P, Ainsworth EA, Pederson T, Moore CE, Brown KL, Raines C, Bernacchi CJ. 2019. High-throughput field phenotyping using hyperspectral reflectance and partial least squares regression (PLSR) reveals genetic modifications to photosynthetic capacity. Remote Sensing of Environment **231**, 111176.
Serbin SP, Dillaway DN, Kruger EL, Townsend PA. 2012. Leaf optical properties reflect variation in photosynthetic metabolism and its sensitivity to temperature. Journal of Experimental Botany **63**, 489-502.
Serbin SP, Singh A, Desai AR, Dubois SG, Jablonski AD, Kingdon CC, Kruger EL, Townsend PA. 2015. Remotely estimating photosynthetic capacity, and its response to temperature, in vegetation canopies using imaging spectroscopy. Remote Sensing of Environment **167**, 78-87.

Sexton T, Sankaran S, Cousins AB. 2021. Predicting photosynthetic capacity in tobacco using shortwave infrared spectral reflectance. Journal of Experimental Botany **72**, 4373-4383.
Silva-Perez V, Molero G, Serbin SP, Condon AG, Reynolds MP, Furbank RT, Evans JR. 2018. Hyperspectral reflectance as a tool to measure biochemical and physiological traits in wheat. Journal of Experimental Botany **69**, 483-496.
Wang S, Guan K, Wang Z, Ainsworth EA, Zheng T, Townsend PA, Li K, Moller C, Wu G, Jiang C. 2021. Unique contributions of chlorophyll and nitrogen to predict crop photosynthetic capacity from leaf spectroscopy. Journal of Experimental Botany **72**, 341-354.
Wu J, Rogers A, Albert LP, Ely K, Prohaska N, Wolfe BT, Oliveira Jr RC, Saleska SR, Serbin SP. 2019. Leaf reflectance spectroscopy captures variation in carboxylation capacity across species, canopy environment and leaf age in lowland moist tropical forests. New Phytologist **224**, 663-674.
Yan Z, Guo Z, Serbin SP, Song G, Zhao Y, Chen Y, Wu S, Wang J, Wang X, Li J, Wang B, Wu Y, Su Y, Wang H, Rogers A, Liu L, Wu J. 2021. Spectroscopy outperforms leaf trait relationships for predicting photosynthetic capacity across different forest types. New Phytologist **232**, 134-147.
Yendrek CR, Tomaz T, Montes CM, Cao Y, Morse AM, Brown PJ, McIntyre LM, Leakey ADB, Ainsworth EA. 2017. High-Throughput Phenotyping of Maize Leaf Physiological and Biochemical Traits Using Hyperspectral Reflectance. Plant Physiology **173**, 614-626.
Zhou J-J, Zhang Y-H, Han Z-M, Liu X-Y, Jian Y-F, Hu C-G, Dian Y-Y. 2021. Evaluating the Performance of Hyperspectral Leaf Reflectance to Detect Water Stress and Estimation of Photosynthetic Capacities. Remote Sensing **13**.
